# Editorial: Role of pyroptosis in neurological disorders and its therapeutic approaches

**DOI:** 10.3389/fnagi.2023.1253644

**Published:** 2023-07-25

**Authors:** Jing Wu, Nelli Mnatsakanyan, Muhuo Ji

**Affiliations:** ^1^Department of Pharmacology, Yale School of Medicine, New Haven, CT, United States; ^2^Department of Cellular and Molecular Physiology, The Pennsylvania State University College of Medicine, Hershey, PA, United States; ^3^Department of Anesthesiology, Second Affiliated Hospital, Nanjing Medical University, Nanjing, China

**Keywords:** pyroptosis, mitochondrial dysfunction, gasdermins, neuroinflammation, cognitive dysfunction, neurological disorders

Pyroptosis is a recently characterized inflammatory form of programmed cell death executed by a family of pore-forming proteins called gasdermins (GSDMs). Upon activation, GSDMs form cell transmembrane pores, which release proinflammatory cytokines and alarmins and cause lytic cell death. Pyroptosis mechanisms generally include canonical caspase-1 pathway, non-canonical caspase-4/5/11 pathway, apoptosis protein caspase-3/8-mediated pathway and granzyme-mediated pathway. Among them, the canonical caspase-1 pathway is the most widely investigated pyroptosis pathway, which occurs through the activation of NOD-, LRR-and pyrin domain-containing protein 3 (NLRP3) inflammasome followed by caspase 1-dependent release of IL-1β and IL-18 proinflammatory cytokines, and by GSDMD-mediated pyroptotic cell death. Accumulating evidence suggests that pyroptosis and its relationship with neuroinflammation are involved in the process of aging as well as the pathogenesis of various neurological disorders, including age-related neurodegenerative diseases, and associated neurocognitive impairment. The aim of this Research Topic is to provide an update on the role of pyroptosis in neurological disorders and its therapeutic approaches.

Two prospective articles in this Research Topic reviewed the mechanisms of pyroptosis, particularly the NLRP3 inflammasome canonical pyroptosis. Li et al. reviewed the current knowledge on the components of NLRP3 inflammasome and its role in pyroptosis and perioperative neurocognitive disorders (PNDs), a group of disorders manifest in relation to surgery and anesthesia. They also summarized current drugs/chemicals and technologies that can potentially inhibit the NLRP3 inflammasomes pyroptosis pathway and treat PNDs. Yuan et al. reviewed the existing evidence that electroacupuncture, which is the most widely used alternative medicine treatment, inhibits NLRP3 inflammasome from different pathways such as ionic flux, mitochondrial dysfunction, and reactive oxygen species (ROS) generation, to treat different neurological diseases, including stroke, Alzheimer's disease (AD), and spinal cord injury (SCI), as well as other inflammatory-related diseases.

Using bioinformatics methods, Shan et al. systematically analyzed the expression of pyroptosis-related genes (PRGs) in different SCI models and associated regulation axis, and found that the canonical NLRP3 inflammasome-mediated PRGs are upregulated in four SCI animal models. They also constructed transcription factors (TFs)–NLRP3/PRGs, miRNAs- Nlrp3/PRGs and lncRNAs/cicrRNAs/mRNAs–miRNA- Nlrp3/PRGs (ceRNA) networks. In addition, they predicted traditional Chinese medicine and small, drug-like molecules with NLRP3/PRGs as potential targets.

Pyroptosis has been demonstrated in different cell types in the central nervous system, including neurons, microglia and astrocytes. Three research articles explored the role of pyroptosis of specific cell types in different neurological diseases and potential therapeutics. Cai et al. observed that NLRP3 inflammasome-mediated pyroptosis is related to the pathogenesis of hippocampal neuronal damage and cognitive dysfunction in AD mice. They further observed that Salidroside, a phenolic glycoside compound that is the main active ingredient extracted from Rhodiola Rose, alleviated Aβ aggregation, Tau hyperphosphorylation, NLRP3-mediated pyroptosis, and neuroinflammation in AD mice. He et al. observed that NLRP3 inflammasome-mediated pyroptosis is involved in antipsychotics-induced astrocyte death in a human astrocyte cell line. Moreover, treatment with a histamine H_1_ receptor agonist, 2-(3-trifluoromethylphenyl) histamine, reduced the antipsychotic-induced activation of NLRP3/caspase-1 signaling and astrocyte death. Finally, Ji et al. found that in an animal model of hypertensive cerebral small vessel disease, treatment with Trimethylamine N-oxide (TMAO), a metabolite of intestinal flora, caused elevated ROS production, NLRP3 inflammasome activation and mitochondrial impairment in the oligodendrocytes, leading to enhanced pyroptosis-related inflammatory death of oligodendrocytes of the cerebrum and demyelination of white matter in corpus callosum regions.

Taken together, studies published in this Frontiers Research Topic highlight the role of pyroptosis in neurological disorders, such as PNDs, AD, and SCI. In addition, these articles investigated drugs that target pyroptosis, particularly the NLRP3 inflammasome canonical pyroptosis pathway, for the treatment of these disorders. We believe that this Research Topic will open new avenues for future studies to reveal the underlining molecular mechanism of pyroptosis, its role in neurological disorders and stimulate the development of novel therapeutic approaches.

## Author contributions

JW prepared the initial draft of the manuscript. JW, NM, and MJ revised the manuscript. All authors contributed to the article and approved the submitted version.

